# A 3D Tissue-Engineering Model of Craniosynostosis to Study the Microenvironmental Signals Leading to Premature Suture Ossification

**DOI:** 10.3390/bioengineering13070746

**Published:** 2026-06-26

**Authors:** Mariangela Meyer, Holmfridur Rist Jonsdottir, Isabel Amado, Javier Gutierrez Gonzalez, Shirley Bracken, Kulwinder Kaur, Tom Hodgkinson, Dylan J. Murray, Arlyng González-Vázquez, Fergal J. O’Brien

**Affiliations:** 1 Tissue Engineering Research Group (TERG), Department of Anatomy and Regenerative Medicine, Royal College of Surgeons in Ireland (RCSI), University of Medicine and Health Sciences, D02 YN77 Dublin, Ireland; marymeyerv@gmail.com (M.M.); holmfridurrist@gmail.com (H.R.J.); newleyamado@gmail.com (I.A.); javiergg@rcsi.ie (J.G.G.); kulwinderkaur@rcsi.ie (K.K.); tomhodgkinson@rcsi.ie (T.H.); 2 Advanced Materials and Bioengineering Research Centre (AMBER), Trinity College Dublin (TCD), D02 PN40 Dublin, Ireland; 3 National Paediatric Craniofacial Centre, Children’s Health Ireland (CHI) at Temple Street, D01 YC67 Dublin, Ireland; shirley.bracken@childrenshealthireland.ie (S.B.); dylanmurray@plasticsurgeon.ie (D.J.M.); 4 School of Pharmacy & Biomolecular Sciences (PBS), Royal College of Surgeons in Ireland (RCSI), University of Medicine and Health Sciences, D02 YN77 Dublin, Ireland; 5 Trinity Centre for Bioengineering, Trinity College Dublin (TCD), D02 PN40 Dublin, Ireland

**Keywords:** non-syndromic craniosynostosis, calvarial sutures, patient-derived model, extracellular matrix, tissue engineering

## Abstract

Craniosynostosis is a congenital bone developmental condition characterized by the premature ossification of calvarial sutures, leading to restricted skull expansion and potential neurological complications. Although little is known about the signaling that governs this accelerated fusion, our research group has previously identified a stiffness-dependent upregulation of osteogenic genes in cells derived from fused sutures, highlighting the role of mechanotransduction in disease progression. Building on these findings, the present study describes the development of a unique patient-derived three-dimensional (3D) tissue-engineering (TE) model of non-syndromic craniosynostosis (NS-CS) to investigate how extracellular matrix (ECM) composition and biochemical cues regulate ossification timing and patterns. Cells isolated from clinically relevant tissues, surgically obtained from patent and prematurely fused calvarial sutures of pediatric NS-CS patients, were characterized and cultured under both two-dimensional (2D) and 3D suture-mimicking conditions. Comparative analysis revealed differences in cellular responsiveness between cells isolated from fused and patent sutures across the different experimental conditions, with cells from fused sutures consistently exhibiting higher expression of osteogenic markers. Notably, the elevated expression of osteogenic and chondrogenic markers suggested the possible involvement of endochondral-like ossification mechanisms during the pathological process of suture fusion. This patient-derived model was designed to recapitulate biophysical and biochemical features of the extracellular matrix of healthy and pathological sutures, serving as a tool for future research, helping us to understand the underlying mechanisms behind the pathophysiology of craniosynostosis.

## 1. Introduction

The morphogenesis of the human calvarium is a complex and highly regulated developmental process that initiates during embryogenesis and is completed during adulthood [1,2,3]. Human calvarial bones arise from a dual embryonic origin, the unsegmented paraxial mesoderm, and the craniofacial neural crest, and are primarily formed through a mesenchymatous phase followed by an intramembranous ossification process [3,4]. Calvarial bone plates are connected and articulated with one another at fibrous joints known as calvarial sutures. These sutures possess key mechanical properties and play a crucial role in facilitating the flexibility of the skull during birth and subsequent expansion of the calvarial vault for proper postnatal brain development [2,5,6,7]. Calvarial sutures are formed by heterogeneous non-ossified mesenchymal tissue, including different cell lineages such as skeletal progenitor cells, mesenchymal cells, osteogenic cells and osteoclasts [8,9,10]. The balance between sutural elasticity, osteogenesis and brain growth ensures healthy morphogenesis of the skull [11,12].

Under pathological conditions, deviation from healthy calvarial development and normal suture patency can occur, resulting in developmental disorders such as craniosynostosis (CS). Craniosynostosis is characterized by the premature ossification of one or more calvarial sutures, which results in abnormal and non-physiological development of craniofacial structures [7,9,13]. Premature fusion of a calvarial suture leads to a reduction in the growth of the adjoining calvarial bone plates and, consequently, a compensatory overgrowth occurs at other sutures. These changes have been correlated with restricted brain growth and associated with increased intracranial pressure, which when untreated, may result in brain damage and neurodevelopmental impairment [7,14,15,16]. To date, craniosynostosis continues to represent a significant medical concern, as there are no pharmacological treatments available, nor early diagnosis methods. Surgical intervention is currently the only option to address the resulting abnormal head shape and its consequences. Various forms of cranial vault remodeling (CVR), ranging from the insertion of springs and distractors to a total CVR, can be associated with major risks and potential complications and with the potential for re-synostosis [7,12,17].

Craniosynostosis can be classified into syndromic or non-syndromic (NS-CS). Among the two different types, the non-syndromic variant is the most common type of craniosynostosis. Unlike the syndromic type, the non-syndromic variant has not been associated with genetic factors and extracranial dysmorphisms, but it has been directly linked to microenvironmental causes [9,13,18,19]. A number of studies have shown that the coordinated expression of several genes—which play crucial roles in key signaling pathways by regulating cell lineage commitment, differentiation, proliferation and apoptosis—is responsible for the development, patency and closure of the calvarial bones and sutures. In particular, alterations in the homeobox gene MSX2, as well as in the FGFR1, FGFR2, FGFR3, EFNB1, ERF, TCF12 and TWIST genes, are known to be responsible for the genetic mechanism associated with the development of craniosynostosis [13,20,21]. Nonetheless, due to a lack of suitable research models, most studies carried out on craniosynostosis base their investigations on targeting specific genetic mutations rather than variations in gene expression levels due to external stimuli from the surrounding environment [22]. Recently, a study carried out by our group has identified the stiffness-dependent upregulation of osteogenic genes in cells isolated from fused sutures [9]. However, the influence of biochemical and biophysical factors on the premature suture ossification observed during craniosynostosis has yet to be investigated.

Since cell populations in the sutures do not have intrinsic proliferation and differentiation potential, they produce new bone at the sutural edges of the bone fronts only in response to signals arising from the neurocranium and dura mater [20,23,24]. However, under pathological microenvironmental effects, alteration in the physiology of the calvarial sutures may occur as a consequence of particular external stimuli such as cyclic loading from muscle activity and traumatic impacts [9,18,19]. It has been demonstrated that mechanical forces applied to the calvarial sutures during development can increase the risk of non-syndromic craniosynostosis by causing changes in their morphology, vascularization and by affecting their native process of osteogenesis [18,25]. Together, this suggests that pathological changes in the physiology of the sutures of children with craniosynostosis may be directly connected to changes in the stimuli provided by the surrounding environment, which might impair the normal functional capacity of cells within the sutures to perceive and respond to these signals.

In order to evaluate if alterations in the biochemical and biophysical extracellular environment are connected to an accelerated suture ossification and the specific signal transduction mechanisms that underpin this pathological behavior, we investigated the role of microenvironmental cues in tissue samples from children diagnosed with non-syndromic craniosynostosis. Although two-dimensional (2D) culture systems are still the most commonly used platforms in cell-based research due to their convenience, easy establishment and low-cost maintenance, 2D culture systems do not mimic the native tissue three-dimensional (3D) matrix structure [15,26]. In our study we overcome these limitations by utilizing tissue-engineering (TE) techniques. We fabricated a 3D single-layered collagen-based culture model compromising bone-mimicking composed of type I microfibrillar collagen (Col I) and nano-hydroxyapatite (nHA), and suture-mimicking scaffolds fabricated combining Col I, type II collagen (Col II) and hyaluronic acid (HyA), which provided strength and flexibility as well as mechanical, physical and biochemical support, creating an ideal 3D niche for cell growth. This 3D culture model was designed to mimic selected biophysical and biochemical features of the extracellular matrix (ECM) of calvarial bones and sutures, enabling comparison of the cellular response across the different 3D scaffolds in combination with osteogenic and chondrogenic culture conditions.

## 2. Materials and Methods

### 2.1. Tissue Collection and Cell Isolation

**Figure 1:**
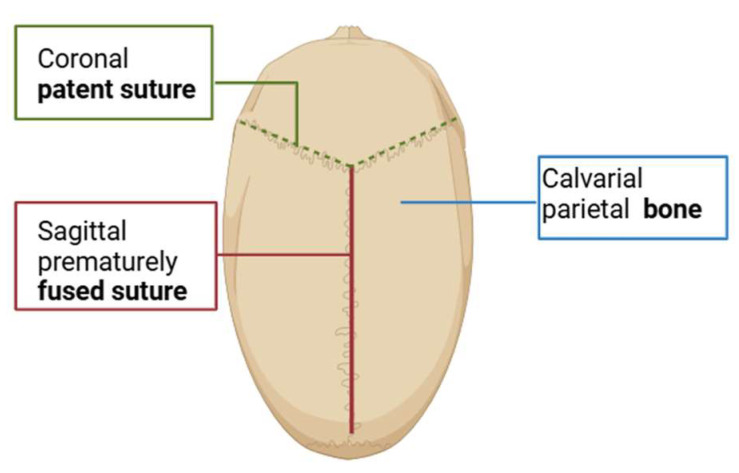
Schematic of the origin of the tissues obtained from sagittal NS-CS patients, unfused coronal patent suture, sagittal prematurely fused suture, and parietal bone as positive control. In patent samples, the term “suture” refers specifically to the intrasutural mesenchymal tissue of the skull, and in fused samples, this term corresponds to prematurely ossified mesenchyme.

Cells were isolated from the three different sample groups: patent (unfused) sutures, fused sutures and calvarial bone, of children 6 months old diagnosed with Sagittal NS-CS, as illustrated schematically in (Figure 1). Samples were obtained from discarded tissues during cranial vault remodeling procedures at Children’s Health Ireland (CHI) at Temple Street. Informed written parental consent was obtained prior to surgery, and all methods were performed in accordance with the relevant guidelines and regulations (ethical approval n°. 19.032 from the Ethics and Research Committee at CHI Temple Street). Cells from each tissue group, patent and fused sutures and calvarial bone were isolated following procedures described previously [9]. Cells were isolated and re-suspended into growth media (GM), consisting of low-glucose Dulbecco’s Modified Eagles medium (DMEM) supplemented with 10% fetal bovine serum (FBS) and 1% penicillin/streptomycin (P/S) cocktail (Sigma-Aldrich, Ireland). Subsequently, cells were seeded at a density of 1 × 10^6^ into T175 flasks and GM was changed every three days. In addition, tissue samples were kept as freshly resected tissue sections in order to characterize the native tissue histologically.

### 2.2. Characterization of Cells Isolated from Patent Sutures, Fused Sutures and Calvarial Bone

#### 2.2.1. Cell Characterization by Flow Cytometry

Flow cytometry analysis was performed to identify the presence of characteristic mesenchymal stem cell (MSC) surface markers, which included: CD90, CD105, CD44, CD73, CD45, CD34, CD11b and major histocompatibility complex Class II molecule (MHC II), using the human MSC (hMSC) Analysis Kit (BD Biosciences, Wokingham, UK) according to the manufacturer’s instructions. Briefly, cell pellets of 5 × 10^5^ cells/mL from the three different sample groups (*n* = 3 per group), were labeled with corresponding antibodies, conjugated with either FITC, phycoerythrin (PE), PerCP-Cy5.5, or allophycocyanin (APC) pigment complex. After staining, the number of cells positive for each marker were quantified with an Attune NxT flow cytometer system and the Attune Cytometric software (Invitrogen, Paisley, UK).

#### 2.2.2. Cell Characterization by Quantitative Real-Time Polymerase Chain Reaction (RT-PCR)

Cells from the three different sample groups (*n* = 3 per group) were initially seeded in 6-well plates at a density of 1 × 10^4^ cells/well and exposed to GM for 7 days. mRNA was isolated from each sample group by adding QIAzol lysis reagent (Qiagen, Manchester, UK) and flash freezing at −80 °C prior analysis. mRNA isolation was performed using the RNeasy Minikit (Qiagen, Manchester, UK) according to the manufacturer’s instructions. Briefly, two-step reverse traNSCSription and Real-Time PCR were performed using Quantitect Reverse TraNSCSription Kits (Qiagen, Manchester, UK) and Sensimix SYBR low Rox PCR Kits (Medical Supply Company, Mulhuddart, Ireland), respectively, loading a total of 2.5 ng of cDNA per reaction. Primer amplification efficiency was compatible with the comparative ΔΔCt method used for expression of the results. The fold induction index was normalized by the expression of a housekeeping gene (18S) for each sample. The PCR was initiated with an activation step of 15 min at 95 °C, followed by 40 cycles of denaturation of 15 s at 94 °C, an annealing cycle of 30 s at 55 °C and finally, an extension cycle of 30 s at 72 °C, followed by a melting curve, in an QuantStudio 3 Real-Time PCR thermocycler (Applied BioSystems, Dublin, Ireland). The panel of primers used for the Real-Time PCR was selected to assess markers indicative of lineage specification and cellular multipotency, and included: JNK3 (Qiagen, QT00006923); SNAI1 (Qiagen, QT00010010); SNAI2 (Qiagen, QT00044128); Nestin (Qiagen, QT00235781); SOX9 (Qiagen, QT00001498); TWIST (Qiagen, QT00011956); SOX2 (Qiagen, QT00237601); SOX10 (Qiagen, QT00055405); NCAM1 (CD56) (Qiagen, QT02457049); ITGβ1 (CD29) (Qiagen, QT00024654); VCAM1 (CD106) (Qiagen, QT00018347), OCT4 (Qiagen, QT02323405) and the housekeeping gene 18S (Qiagen, QT00199367).

#### 2.2.3. Determination of Cell Intrinsic Osteogenic Potential Through ALP Activity and Calcium Production

Cells isolated from the three different sample groups (*n* = 3 per group) were cultured in monolayer at a density of 3 × 10^4^ cells/mL in 6-well plates and exposed to growth and osteogenic media (OM) for 21 days. The OM consisted of GM supplemented with 100 nM dexamethasone, 50 μg/mL ascorbic acid and 10 mM β-glycerophosphate (Sigma Aldrich, Arklow, Ireland). The osteogenic potential was analyzed in terms of alkaline phosphatase (ALP) activity using the SensoLyte pNPP Alkaline Phosphatase Assay Kit (AnaSpec, CA, USA) and in terms of extracellular matrix mineralization based on calcium deposition levels, as quantified by a Calcium (CPC) LiquiColour Kit (Stanbio Laboratory, TX, USA). Assays were carried out according to the manufacturer’s protocols. ALP activity and mineralization results were subsequently determined using a standard curve and normalized against total dsDNA content per sample. dsDNA was measured using the Invitrogen Quant-iT PicoGreen dsDNA kit (BioSciences, Dublin, Ireland).

#### 2.2.4. Determination of Cell Intrinsic Chondrogenic Potential Through Sulphated Glycosaminoglycan (sGAG) Deposition

Cells from the three different sample groups (*n* = 3 per group) at passage 2 (P2), were cultivated as pellets in 15 mL falcon tubes at a density of 2.5 × 10^5^ cells/pellet and exposed for 21 days to GM and to incomplete (ICM) and complete chondrogenic media (CCM). The ICM consisted of high glucose serum-free DMEM supplemented with 1% penicillin/streptomycin (P/S) cocktail, 100 nM dexamethasone, 50 μg/mL ascorbic acid, 40 µg/mL Proline (Sigma Aldrich, Arklow, Ireland) and 10 µL/mL Insulin Transferrin Selenium (ITS) supplement (BD Biosciences, Wokingham, UK) while the CCM corresponded to ICM additionally supplemented with 20 ng/mL Human TGFβ-3 (Peprotech, London, UK). To assess the chondrogenic potential, the sGAG deposition was quantified using a Blyscan sGAG assay kit (Biocolor Life Sciences Ltd., Belfast, UK), as per the manufacturer’s instructions. sGAG concentration was normalized against the dsDNA content per sample as previously described.

### 2.3. Fabrication of the 3D Culture Scaffolds

To fabricate the collagen-based scaffolds, a freeze-drying method previously described and standardized within our research group was used [27].

#### 2.3.1. Bone-Mimicking Scaffolds

The bone-mimicking scaffolds were fabricated according to the technique developed and standardized in our lab [28,29] and consisted of type I microfibrillar collagen (Col I) derived from bovine Achilles tendon (Collagen Matrix, NJ, USA) in combination with nano-hydroxyapatite (nHA) particles formulated in-house. To fabricate the collagen-based scaffolds, a freeze-drying method previously described and standardized within our research group was used [30]. Briefly, 1.8 g of Col I were blended with 0.5 M acetic acid solution (pH 2.8) for 90 min at 4 °C, in a cooled reaction vessel using an IKA Ultra Turrax T15 overhead blender (IKA Works Inc., NC, USA) at a speed of 15,000 rpm. The nHA suspension was prepared by adding 1.47 g CaCl_2_, 2.27 g Na_3_PO_4_, 0.02 g NaOH and 200 µL Darvan to 200 mL of dH_2_O. This suspension was incorporated into the blended Col I suspension in 10 mL aliquots every 20 min to yield a slurry with final concentration of 1:1 *w*/*w* Col I:nHA. The resulting Col l—nHA slurry was degassed in a vacuum chamber at a pressure of 50 mTorr. Subsequently, 0.3 mL of slurry was pipetted into 10 mm cylindrical stainless-steel molds and freeze-dried (Virtis Genesis 25EL, Biopharma, Winchester, UK) at a constant cooling rate of 1 °C/min to a final temperature of −40 °C. Following freeze-drying, the scaffolds were dehydrothermally (DHT) crosslinked for 24 h at 0.05 bar pressure and 105 °C in a vacuum oven (VacuCell, MMM, Planegg, Germany). The scaffolds were hydrated in PBS (Sigma Aldrich, Arklow, Ireland) before being chemically crosslinked for 2 h using a solution of 1-ethyl-3–3-dimethyl aminopropyl carbodiimide (EDAC)/N-hydroxysuccinimide (NHS) (Sigma–Aldrich, Arklow, Ireland), in the ratio 5:2 EDAC:NHS [27].

#### 2.3.2. Suture-Mimicking Scaffolds

The suture-mimicking scaffolds were fabricated according to the method previously developed and standardized within our laboratory [27,31,32,33]. These scaffolds consisted of microfibrillar Col I in combination with type II collagen (Col II) isolated from porcine knee cartilage (Symatese, Vourles, France) and hyaluronic acid (HyA) sodium salt derived from *Streptococcus equi* (molecular weight 1500–1800 kDa) (Sigma-Aldrich, Arklow, Ireland). The Col I/Col II—HyA slurry consisted of both Col I and Col II at a ratio of 1:1, with a total collagen concentration of 0.5% (*w*/*v*), in the presence of 0.05% (*w*/*v*) HyA. Briefly, the Col I/Col II suspension was prepared by blending 0.9 g Col I and 0.9 g Col II in 0.5 M acetic acid for 90 min at a speed of 15,000 rpm. Simultaneously, 0.16 g HyA was dissolved in 0.5 M acetic acid and subsequently added drop by drop into the Col/Col II suspension. Following blending, the resulting Col I/Col II—HyA slurry was degassed in a vacuum chamber and underwent a freeze-drying cycle at −20 °C at a freezing rate of −1 °C/min, in 10 mm cylindrical stainless-steel molds. Scaffolds were crosslinked and sterilized using a dehydrotheramal treatment and chemically cross-linked with a solution of EDAC:NHS in the ratio 5:2 [31,32,33].

### 2.4. Cell Seeding on 3D Scaffolds

Cells were harvested from calvarial bone, fused sutures and patent suture tissues and isolated as mentioned beforehand. Before seeding on 3D scaffolds, cells were exposed to complete chondrogenic media (CCM) and osteogenic media (OM), generating a pre-exiting chondrogenic and osteogenic environment. Cells cultured in (OM) are seeded on both bone- and suture-mimicking scaffolds exposed to (GM) and (OM). Cells cultured in (CCM) are seeded on both bone- and suture-mimicking scaffolds exposed to (GM), (ICM) and (CCM).

### 2.5. Characterization of Scaffolds

#### 2.5.1. Evaluation of the Porous Architecture of 3D Scaffolds Using Scanning Electron Microscopy (SEM)

The different porous microarchitecture of the bone- and suture-mimicking scaffolds was examined by sagittal sectioning the 3D scaffolds through their frontal plane and assessed using scanning electron microscopy (SEM). Samples were sputter-coated with gold and inserted into the chamber of a Supra Variable Pressure Field Emission Scanning Electron Microscope (Zeiss, Oberkochen, Germany). Micrographs were then captured using SmartSEM software (Zeiss, Oberkochen, Germany). SEM imaging was used to qualitatively assess differences in scaffold architecture. Quantitative analysis of the pore size has been previously performed and validated in earlier studies carried out in our lab: Col I—nHA scaffolds have a pore size of 136 μm and 99% of porosity while Col I/Col II—HyA scaffolds have a pore size of 155 ± 5.65 μm and 99% of porosity [27].

#### 2.5.2. Assessment of ALP Activity and Calcium Production

Expression of osteogenic markers was biochemically measured in the three different sample groups cultured on the bone- and suture-mimicking scaffolds at a density of 3.5 × 10^5^ cells/scaffolds, when exposed to growth, osteogenic and chondrogenic media for 21 days. The osteogenic potential of these cells was assessed by measurement of calcium deposition in the extracellular matrix using the Calcium (CPC) LiquiColour Kit (Stanbio Laboratory, TX, USA) and in terms of ALP activity, by using the SensoLyte pNPP Alkaline Phosphatase Assay Kit (AnaSpec, CA, USA), according to the manufacturer’s instructions.

#### 2.5.3. Assessment of GAG Deposition

A Blyscan GAG assay kit (Biocolor Life Sciences Ltd., Belfast, UK), was used to measure the quantity of GAG laid down on the bone- and suture-mimicking scaffolds. As described in the previous section, cells were cultured on the bone- and suture-mimicking scaffolds at a density of 3.5 × 10^5^ cells/scaffolds and incubated in growth, osteogenic and chondrogenic media for 21 days. Once the incubation time was completed, assessment of GAG deposition was carried out, accessing chondrogenic differentiation, as GAGs are a major component in cartilage ECM.

#### 2.5.4. Histological Analysis of ECM Synthesis by Cells on 3D Scaffolds

Cells were incubated on bone- and suture-mimicking scaffolds as described in the above section. Scaffolds were fixed in 10% formalin for 12 h at 4 °C and cryopreserved by incubating in a 30% sucrose solution for 48 h. Scaffolds were then transferred into plastic molds and embedded into cryopreservative media for frozen specimens to ensure optimum cutting temperature (OCT compound) (Fisher-Scientific, Dublin, Ireland). Subsequently, they were rapidly frozen in dry ice. Embedded scaffolds were then serially sectioned at −20 °C using a cryostat machine (LEICA CM 1950, Leica Microsystems, Hesse, Germany) to obtain slices of 10 μm thickness. Sections were successively mounted on Superfrost Plus slides^TM^ (Fisher-Scientific, Dublin, Ireland), let to air-dry overnight and hydrated with Dulbecco’s Phosphate-Buffered Saline (PBS) before staining. Sections were stained with haematoxylin and eosin (H&E) (Sigma-Aldrich, Arklow, Ireland) to reveal the distribution of cells throughout the scaffolds and with toluidine blue (Sigma Aldrich, Arklow, Ireland) which stains sGAG. In addition, ALP (Sigma-Aldrich, Arklow, Ireland) and 2% Alizarin Red (ScienCell, CA, USA) staining were used to evaluate osteogenic ECM deposition.

### 2.6. Histological Analysis of Native Tissues

Tissue samples from patent sutures, fused sutures and calvarial bone were preserved as freshly resected sections by fixing in 10% formalin and 30% sucrose solution directly after being removed during surgery. By following the steps described in the previous section, tissue samples were embedded into OCT compound and serially sectioned at −20 °C using a cryostat machine (LEICA CM 1950, Leica Microsystems, Hesse, Germany) to obtain tissue slices of 5 μm thickness. Tissue sections were mounted on Superfrost Plus slides^TM^ and stained with H&E. Histological assessment of cartilaginous tissue based on GAG deposits was performed using 0.025% toluidine blue staining. ECM mineralization was analyzed by calcein blue staining and ALP activity was determined by Naphthol AS-MX phosphate and Fast red TR Salt (Sigma-Aldrich, Arklow, Ireland) assessment.

### 2.7. Data Processing and Statistical Analysis

Data processing and statistical analysis were performed using the GraphPad Prism software (version 8, GraphPad Software Inc., CA, USA). Statistical significance was determined using a two-way analysis of variance (ANOVA) followed by Tukey’s post hoc analysis for multiple comparisons. Results were expressed as the mean ± SEM, unless stated otherwise, and considered statistically significant for *p*-values less than or equal to 0.05 (*p* ≤ 0.05). All experiments were performed for 3 donors (*n* = 3), each with 3 experimental repeats.

## 3. Results

### 3.1. Cells from Patent Sutures Retain Their Lineage-Specificity and Show Higher Multilineage Differentiation Potential than Fused Sutures

**Figure 2:**
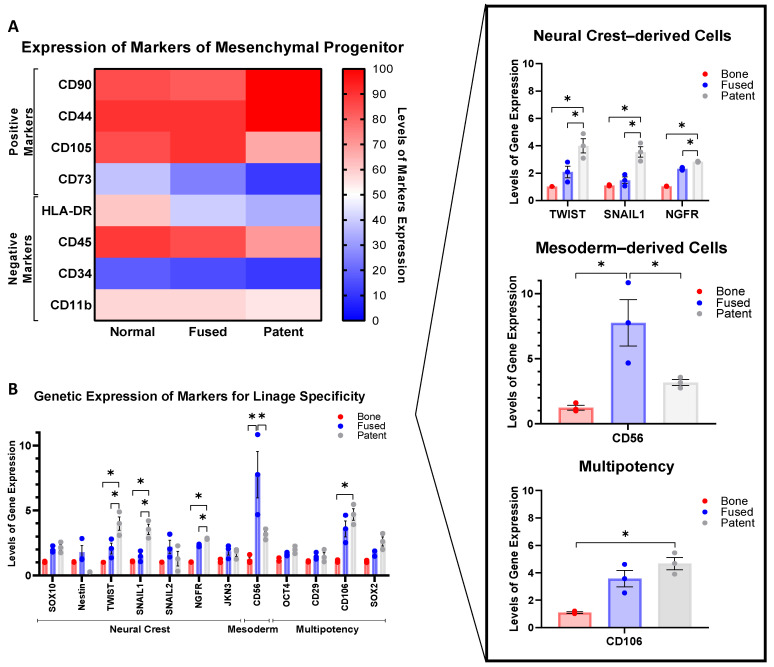
Evaluation of the native stemness and lineage-specificity of cells from calvarial bone and fused and patent sutures. (**A**) Heatmap representing the percentage of cells expressing positive and negative markers of mesenchymal progenitor, as measured by flow cytometry after 7 days in growth media (GM). (**B**) RT-PCR data showing the genetic expression of markers for neural crest- and mesoderm-derived cells, and multipotency, after 7 days in GM. Closeup showing significant differences in gene expression levels according to groups of interest. Donors N = 3; technical repeats *n* = 3. Data is presented as mean ± SEM. Two-way ANOVA with Tukey’s Multiple Comparisons Test: * *p* < 0.05, ** *p* < 0.01.

In order to investigate the native stemness of cells isolated from calvarial bone and patent and fused sutures of children diagnosed with NS-CS, flow cytometry was utilized to assess the expression profile of characteristic surface markers of mesenchymal progenitors (Figure 2A).

Results showed that despite the visible variability in the expression levels between the three different sample groups, levels of positive mesenchymal progenitor surface markers (CD90 and CD44) were higher in cells from patent sutures compared to levels found in calvarial bone and fused sutures (Figure 2A). It was also observed that the lowest expression levels of markers targeting hematopoietic cells (CD34), MHC molecules (HLA-DR) and immune cells (CD45 and CD11b) were detected on cells from patent sutures. Different levels of stemness between the three sample groups are also reflected by the differences in their expression profile. Cells from patent sutures conserve the strongest mesenchymal progenitor poperties and are therefore capable of more rapid regeneration, with reduced expression in all negative contols, immune (CD45a and CD11b) and heamtopoietic markers (CD34), whilest maintining stong expression in positive mesenchymal progenitor surface markers.

In addition, when the sample groups of calvarial bone, fused and patent tissue were investigated (Figure 2B), each tissue group maintained a distinct set of surface markers, therefore preserving their intrinsic lineage-specificity. Considering that calvarial bones arise from the paraxial mesoderm and coronal suture cells originate from the neural crest, we determined the genetic expression profile of a customized panel of characteristic markers of neural crest- and mesoderm-derived cells as well as multipotency markers by Quantitative Real-Time Polymerase Chain Reaction (RT-PCR). Findings revealed significant upregulation of the expression levels of markers targeting neural crest–derived cells (TWIST1, SNAIL1 and NGFR) and multipotency (CD106) on cells from patent sutures, while CD56, a characteristic marker of mesoderm-derived cells, was upregulated in cells from fused sutures. Interestingly, low expression levels of CD56 were found in cells from calvarial bone. These results confirmed that cells from patent sutures conserve their intrinsic lineage-specificity and have the higher multilineage differentiation potential, contrary to what was observed in cells from fused sutures.

### 3.2. Calvarial Bone, Fused Suture and Patent Suture Tissues Showed Individualized Distinct Morphology, with Enhanced Mineralization in Fused Sutures

**Figure 3:**
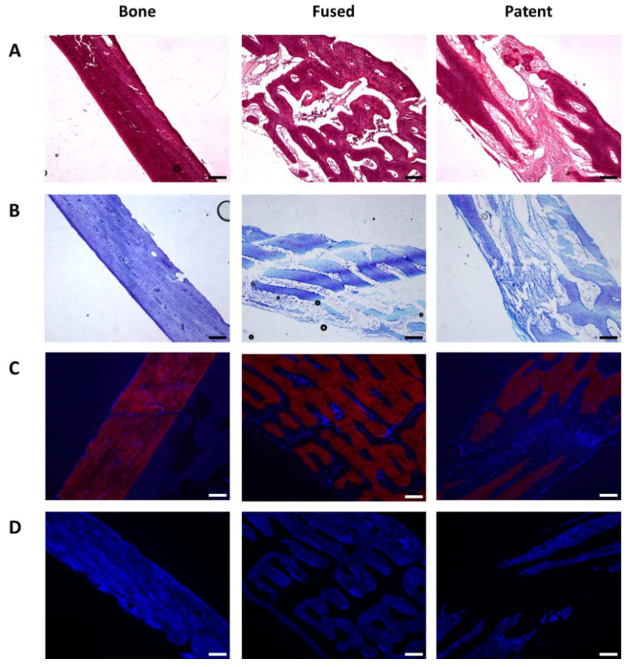
Histological analysis of freshly resected calvarial bone, fused sutures and patent sutures. Brightfield imaging of (**A**) haematoxylin and eosin (H&E) staining displaying tissue morphology and (**B**) toluidine blue (TB) staining, specific for glycosaminoglycans (GAGs). Immunofluorescence imaging of (**C**) alkaline phosphatase (ALP) staining (red) and (**D**) calcein blue staining showing mineralization levels (blue). Scale bar = 200 µm. Donors N = 3; technical repeats *n* = 3.

Tissue examination of freshly resected samples from patent sutures, prematurely fused sutures and calvarial bone were evaluated histologically in order to characterize the native components of the in vivo tissue microenvironment (Figure 3).

H&E analysis of post-surgical sections revealed dense, bone-like tissue in calvarial bone samples, whereas patent and fused sutures contained fibrous, cartilage-like tissue, with higher proportion in patent sutures than in the fused tissues (Figure 3A). This pattern was also confirmed by toluidine blue (TB) staining (Figure 3B) which revealed higher expression of GAGs on sections from patent sutures.

**Figure 4:**
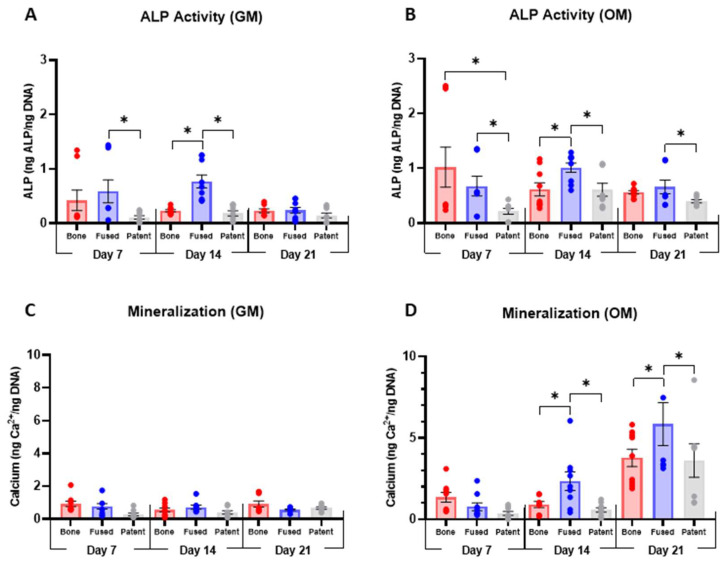
Evaluation of the intrinsic osteogenic potential of cells from patent and fused sutures by means of measurement of alkaline phosphatase (ALP) activity and calcium deposition, in comparison with cells from calvarial bone. (**A**,**B**) ALP activity of cell populations cultured in growth media (GM) and in osteogenic media (OM) after 7, 14 and 21 days in culture. (**C**,**D**) Mineralization of cell populations in GM and in OM after 7, 14 and 21 days in culture measured by means of calcium deposition. Donors N = 3; technical repeats *n* = 3. Data is presented as mean ± SEM. Two-way ANOVA with Tukey’s Multiple Comparisons Test: * *p* < 0.05.

In addition, high ALP activity on fused sutures compared to ALP activity on patent sutures indicated increased mineralization in the prematurely fused sections (Figure 3C). Tissue mineralization was also evaluated based on calcium deposits as determined by calcein blue (CB) staining, which confirmed that fused sutures exhibited higher calcium deposition than patent sutures (Figure 4D). Interestingly, when ALP activity and calcium deposition were compared in fused sutures (Figure 4C,D) a wider distribution of ALP when compared against calcium was observed, which indicated that fused suture sections are still in the early stage of bone formation.

### 3.3. Cells Isolated from Fused Sutures Have the Strongest Osteogenic Response When Exposed to Osteoinductive Growth Factors

To understand the intrinsic osteogenic potential of cells isolated from calvarial bone and patent and fused sutures of children with NS-CS, we evaluated their differentiation potential into an osteogenic phenotype while exposed to both growth and osteogenic media. Expression of osteogenic markers, including ALP activity and calcium deposition, were measured after 7, 14 and 21 days in culture.

Data showed that when cultured with growth media (GM), cells from patent sutures exhibited the lowest ALP activity—compared to cells from fused sutures and normal calvarial bone—at each time point (Figure 4A). On the other hand, levels of mineralization remained consistently low throughout the sample groups during the three different evaluated time points (Figure 4C). In contrast, when samples were cultured in osteogenic media, cells from fused sutures presented significantly higher ALP activity and higher calcium release than cells from patent sutures, reaching the peak of expression after 21 days in culture (Figure 4B,D). Interestingly, after 7 days in OM, ALP activity and calcium release of cells from fused sutures was similar to the levels obtained from cells from the normal calvarial bones—used as a control for fully differentiated osteoblasts—indicating that cells from fused sutures might be able to differentiate towards an osteogenic phenotype earlier than cells from patent sutures.

### 3.4. In 2D Culture, Cells Isolated from Fused and Patent Sutures Showed a Significant Enhanced Chondrogenic Response When Exposed to Chodroinductive Growth Factors

**Figure 5:**
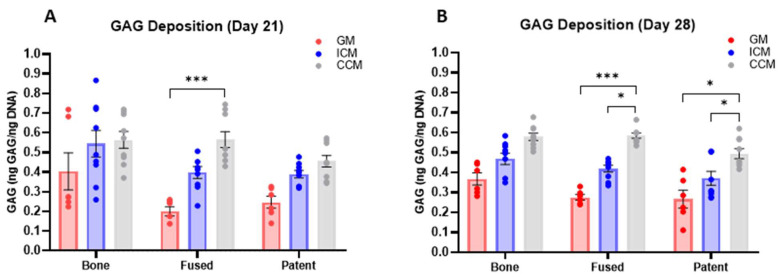
Assessment of the intrinsic chondrogenic potential of cells isolated from patent and fused sutures by means of measurement of sulphated glycosaminoglycan (GAG) deposition, in comparison with cells from calvarial bone. (**A**) GAG levels produced by cells from calvarial bone, fused sutures and patent sutures, cultured for 21 days in growth media (GM), incomplete chondrogenic media (without TGF-β3) (ICM) and in complete chondrogenic media (with TGF-β3) (CCM). (**B**) GAG levels produced by cells from calvarial bone, fused sutures and patent sutures, cultured for 28 days in GM, ICM and in CCM. Donors N = 3; technical repeats *n* = 3. Data is presented as mean ± SEM. Two-way ANOVA with Tukey’s Multiple Comparisons Test: * *p* < 0.05; ** *p* < 0.01; *** *p* < 0.001.

We analyzed the effect of chodroinductive factors on the intrinsic chondrogenic differentiation capacity of cells isolated from calvarial bone and patent and fused sutures. Cells from the three different sample groups were cultivated as pellets for 21 and 28 days and induced to a chondrogenic lineage using two different culture media, incomplete (ICM) and complete chondrogenic media (CCM) (Figure 5). Chondrogenic differentiation was quantitatively assessed by measuring sulphated glycosaminoglycan (GAG) deposition, a major component of the cartilage ECM.

After 28 days of supplementation with TGF-β3, a significantly increased GAG deposition was observed on cells from both fused and patent sutures when cultured in ICM media, compared to GAG deposition found in GM and ICM (Figure 5B). These findings confirmed that the chondrogenic potential of cells from the calvarial sutures significantly depended on the chondroinductive factors present in the microenvironment. Results also suggested that cells from fused sutures are not only able to differentiate faster towards an osteogenic phenotype compared to cells from patent sutures, but also have the ability to commit faster towards a chondrogenic lineage.

### 3.5. Tissue-Engineered Bone and Suture-Mimicking Scaffolds Displayed Distinct Microarchitectural Features Which Supported Cell Distribution Throughout the Culture Surface

**Figure 6:**
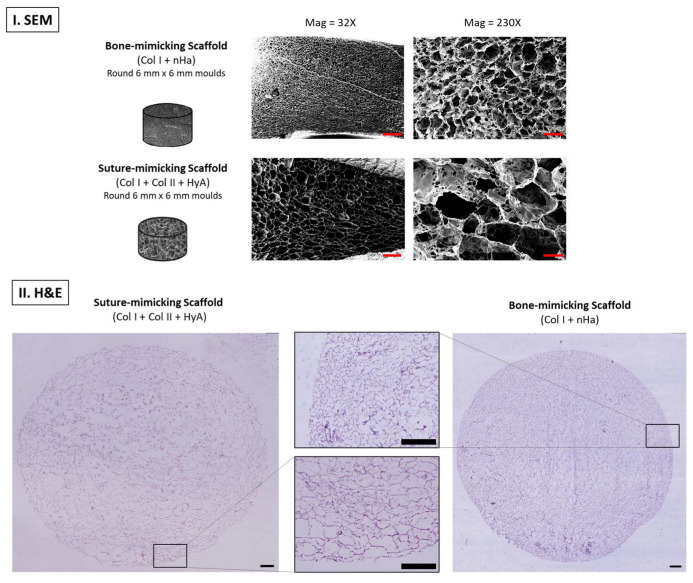
(**I**) Vertical cross-sectional images of the bone-mimicking scaffolds (Col I + nHA) and suture-mimicking scaffolds (Col I + Col II + HyA) obtained by Scanning Electron Microscopy (SEM) at different magnification (32×, Scale bar = 400 µm and 230×, Scale bar = 60 µm). (**II**) Horizontal cross-sectional images of bone- and suture-mimicking scaffolds stained by H&E, highlighting cell migration after 21 days in culture. Scale bar = 500 µm, technical repeats *n* = 3.

In order to emulate the physical and biological properties of the native microenvironment of the human skull, two different types of scaffolds were fabricated as analogs of the calvarial extracellular matrix (ECM) (Figure 6I). Bone-mimicking scaffolds were composed of type I microfibrillar collagen (Col I) and nano-hydroxyapatite (nHA), while suture-mimicking scaffolds were fabricated combining Col I, type II collagen (Col II) and hyaluronic acid (HyA).

The SEM analysis revealed a distinct difference in the microstructural architecture between bone-mimicking scaffolds and suture-mimicking scaffolds (Figure 6I). The bone-mimicking scaffolds exhibited a denser structure with smaller pores compared to a more relaxed structure with larger pores of the suture-mimicking scaffolds. These microstructural differences in the pore architecture have been reported to enhance osteo- and chondrogenesis, respectively [28,33].

Histological analysis via H&E staining further confirmed the distinct architecture observed in bone and suture-mimicking scaffolds when analyzed by SEM. H&E staining of cell-seeded 3D scaffolds maintained in culture for 21 days also showed cells distributed along the scaffolds, especially towards the periphery, suggesting that these scaffolds provided an optimal structural support for cell attachment and subsequent cell proliferation and differentiation.

### 3.6. Cells from Fused Sutures Are the Most Sensitive to Osteoinductive Growth Factors, When Cultured on Suture-Mimicking Scaffolds

**Figure 7:**
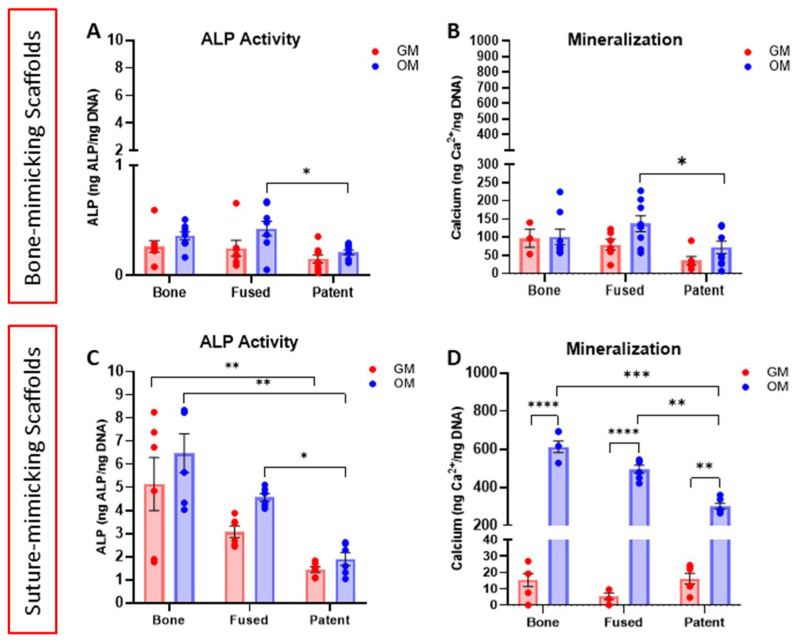
Assessment of the 3D osteogenic potential of cells from patent and fused sutures when cultured for 21 days on bone- and suture-mimicking scaffolds, by means of measurement of alkaline phosphatase (ALP) activity and mineralization in comparison with cells from calvarial bone used as a control. (**A**,**B**) ALP activity and mineralization levels of cell populations cultured in growth media (GM) and in osteogenic media (OM) on bone-mimicking scaffolds. (**C**,**D**) ALP activity and mineralization levels of cell populations cultured in GM and OM on suture-mimicking scaffolds. Donors N = 3; technical repeats *n* = 3. Data is presented as mean ± SEM. Two-way ANOVA with Tukey’s Multiple Comparisons Test: * *p* < 0.05; ** *p* < 0.01; *** *p* < 0.001; **** *p* < 0.0001.

After confirmation that the bone- and suture-mimicking scaffolds served as a suitable structural support for cell functions, they were used to culture cells isolated from calvarial bone and patent and fused sutures in order to evaluate their differentiation potential into an osteogenic phenotype while exposed to both osteo- and chondrogenic microenvironments (Figure 7). Data showed higher intrinsic alkaline phosphatase (ALP) activity and calcium deposition in cells from fused sutures compared to cells from patent sutures when cultured on either bone- or suture-mimicking scaffolds (Figure 7A,D).

In line with results from 2D culture studies (Figure 4), the addition of osteogenic growth factors significantly potentiated ALP activity and mineralization in cells from fused sutures when compared to patent sutures—in both types of scaffolds (Figure 7A–D). However, an up to five-times increase in ALP activity and mineralization was found in cells from fused sutures when cultured on suture-mimicking scaffolds (Figure 7C,D) compared to their response on bone mimicking scaffolds (Figure 7A,B). These findings confirmed enhanced osteogenic activity on cells from fused sutures, even after being exposed to a cartilaginous ECM.

**Figure 8:**
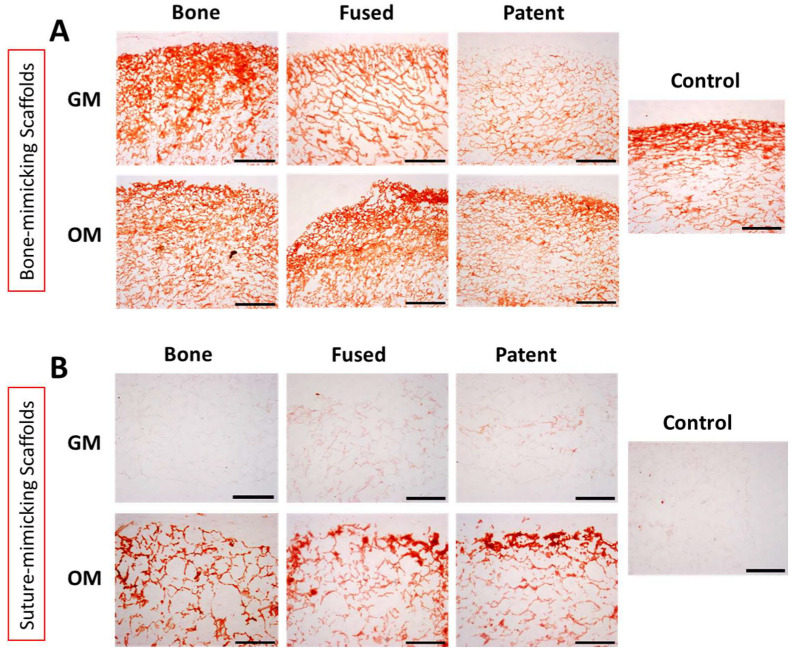
Horizontal cross-sections of cell seeded on (**A**) bone-mimicking scaffold and on (**B**) suture-mimicking scaffold, stained with Alizarin Red (AR), specific for calcium deposition, after 21 days in growth media (GM) and osteogenic media (OM).Cell-free scaffolds were used as control, respectively. Scale bar = 500 µm.

The presence of mineral calcification within the cell-seeded bone- and suture-mimicking scaffolds was also verified by Alizarin Red (AR) staining (Figure 8), with the staining results supporting the biochemical assessment presented in Figure 7. Results corroborated an enhanced osteogenic response to OM compared to GM, especially when cultured on a cartilage-mimicking microenvironment, supporting findings of a significant increase in ECM mineralization shown in Figure 7. Data together suggested that cells from fused sutures are highly sensitive to the surrounding microenvironmental signals and retain a stronger osteogenic phenotype under the provided experimental conditions.

### 3.7. Cells from Calvarial Bone and Sutures Showed Higher Chondrogenic Activity When Cultured in the 3D Suture-Mimicking Scaffolds and Simultaneously Exposed to Osteogenic Growth Factors

**Figure 9:**
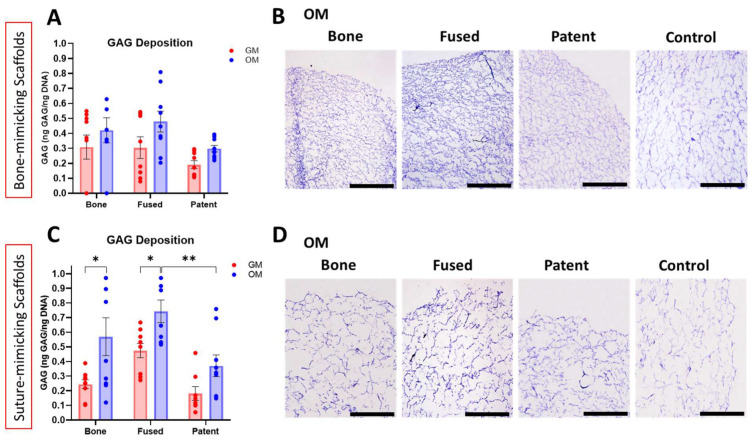
Evaluation of the 3D chondrogenic potential of cells from calvarial bone and sutures tissues when cultured for 21 days on bone- and suture-mimicking scaffolds, by means of measurement of glycosaminoglycan (GAG) deposition. (**A**,**C**) GAG levels produced by cells exposed to growth media (GM) and osteogenic media (OM). Donors N = 3; technical repeats n = 3. Data is presented as mean ± SEM. Two-way ANOVA with Tukey’s Multiple Comparisons Test: * *p* < 0.05; ** *p* < 0.01 (**B**,**D**) Brightfield images of toluidine blue (TB) staining specific for GAGs, on cells seeded with OM. Scale bar = 500 µm.

After assessing the role of bone-like ECM alone and in combination with biochemical factors on the different NS-CS derived cells, the next step was to evaluate the chondrogenic capacity of those cells and the role of suture-mimicking ECM on driving suture patency while being stimulated with osteogenic factors (Figure 9). Their intrinsic chondrogenic potential was quantitatively assessed by measuring GAG deposition and by TB staining.

Findings indicated that cells from fused sutures have the highest production of GAGs in either of the two studied scaffolds (Figure 9A,C). These results were corroborated by TB staining, which revealed that in both bone0 and suture-mimicking scaffolds, cells from fused suture present the greater GAG deposition compared to bone and patent samples (Figure 9B,D). Nevertheless, quantitative assessment showed that when cells from fused sutures are cultured on suture-mimicking scaffolds, a significant increase in GAG expression occurred after being exposed to OM, compared to GAG expression with GM (Figure 9C). This response indicated that osteogenic growth factors promote the expression of cartilage matrix markers under the tested experimental conditions. Notably, cells isolated from fused sutures displayed a stronger response to osteogenic growth factor exposure, suggesting that they might be intrinsically more sensitive to the biochemical than to the biophysical cues or structural properties of the ECM.

### 3.8. Cells from Calvarial Bone and Sutures Showed an Increased Sensitivity to Chondrogenic Growth Factors in Response to the 3D Chondroinductive Culture Conditions

**Figure 10:**
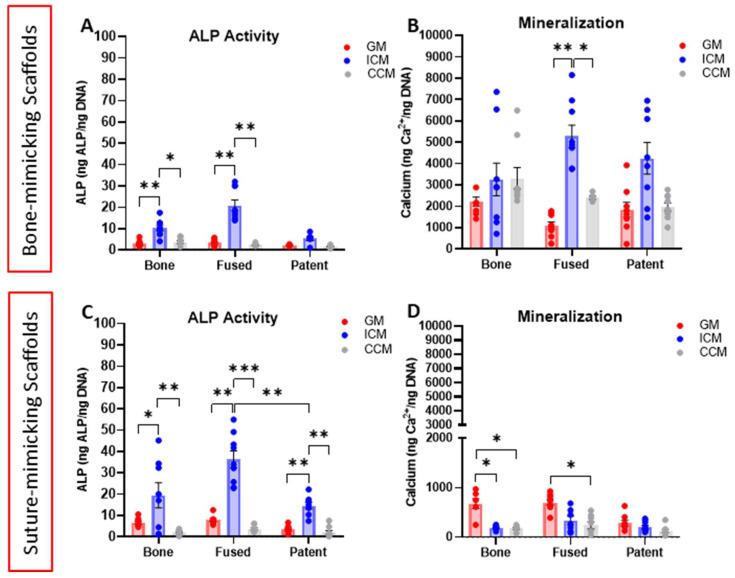
Assessment of the 3D chondrogenic potential of cells from patent and fused sutures by means of measurement of alkaline phosphatase (ALP) activity and calcium deposition in comparison with cells from calvarial bone. (**A**,**B**) ALP activity and mineralization levels of cell populations cultured in growth media (GM), and chondrogenic media, with (CCM) and without TGF-β3 (ICM) on bone-mimicking scaffolds. (**C**,**D**) ALP activity and mineralization levels of cell populations cultured in GM, ICM and CCM on suture-mimicking scaffolds. Donors N = 3; Technical repeats *n* = 3. Data is presented as mean ± SEM. Two-way ANOVA with Tukey’s Multiple Comparisons Test: * *p* < 0.05; ** *p* < 0.01; *** *p* < 0.001.

To complete the last piece of the puzzle that will help us understand the role of specific signals given by the surrounding microenvironment in the differentiation patterns of cell in NC-CS tissues, we evaluated their differentiation potential into an osteogenic phenotype while supported by specific bone and cartilage ECM and exposed to chondrogenic factor (Figure 10). Osteogenic differentiation was quantitatively assessed by measuring ALP activity and calcium deposition on cells cultured on bone- and suture-mimicking scaffolds and exposed for 21 days to GM and chondrogenic media, with and without TGF-β3.

**Figure 11:**
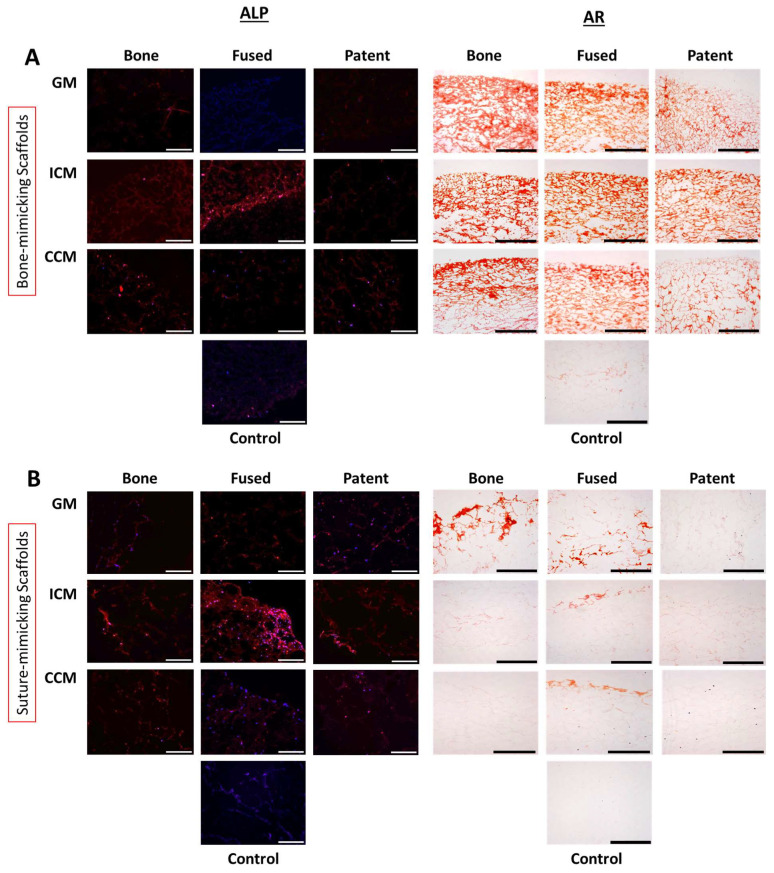
Histological analysis of mineralization in horizontal cross-sections of (**A**) bone-mimicking scaffolds and (**B**) suture-mimicking scaffolds after 21 days in culture in growth media (GM) and incomplete (ICM) and complete chondrogenic media (CCM). ALP: Brightfield images of alkaline phosphatase (ALP) staining, showing increased expression in suture mimicking scaffold. Scale bar = 200 µm. AR: Brightfield images of Alizarin Red (AR) staining displaying calcium deposits. Scale bar = 500 µm.

A significantly higher ALP activity was observed in cells from fused sutures when seeded on suture-mimicking scaffolds compared to cells from patent sutures (Figure 10A,C). Interestingly, in both cases, cells exposed to ICM were those that presented greater ALP activity. On the contrary, mineralization analysis showed that cells from fused sutures cultured on bone-mimicking scaffolds have higher calcium deposition than those seeded on suture-mimicking scaffolds, when exposed to chondrogenic growth factors (Figure 10B,D). ALP activity and calcium deposition patterns were also supported by ALP and AR stainings, which yielded similar results (Figure 11A,B) and corroborated the trends showed in Figure 10.

These findings together suggest that in the absence of osteogenic growth factors, a cartilaginous microenvironment alone does not trigger the expression of osteogenic markers nor promote mineralization of the ECM. This data showed that cells from fused sutures are highly sensitive to the surrounding biochemical factors and can be quickly influenced negatively towards premature ossification.

### 3.9. Cells from Calvarial Bone and Sutures Showed an Enhanced Sensitivity to Chondrogenic Growth Factors, When Cultured on a 3D Chondroinductive Microenvironment

As a final assessment, we evaluated the 3D chondrogenic potential of cells from the three different sample groups by measurement of GAG deposition, when seeded on specific bone and cartilage ECM and exposed to chondrogenic factor.

**Figure 12:**
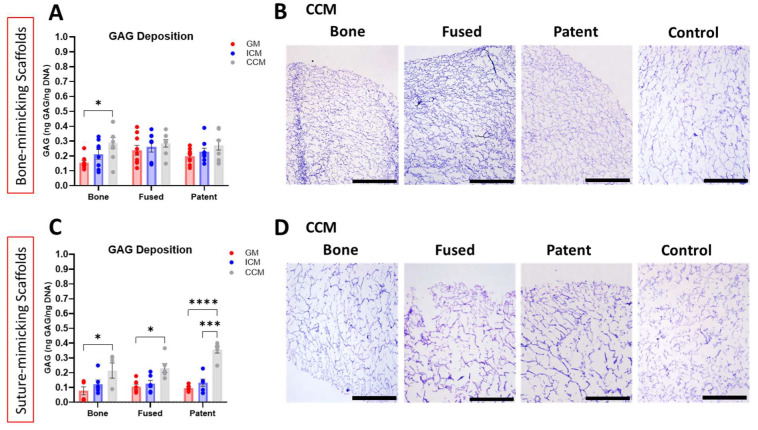
Evaluation of the 3D chondrogenic potential of cells isolated from calvarial bone and suture tissues when cultured for 21 days on bone and suture-mimicking scaffolds, by means of measurement of glycosaminoglycan (GAG) deposition. (**A**,**C**) GAG levels produced by cells exposed to growth media (GM) and incomplete (ICM) and complete chondrogenic media (CCM) with and without TGF-β3, respectively) while seeded on bone- and suture-mimicking scaffolds. Donors N = 3; technical repeats *n* = 3. Data is presented as mean ± SEM. Two-way ANOVA with Tukey’s Multiple Comparisons Test: * *p* < 0.05; ** *p* < 0.01; *** *p* < 0.001; **** *p* < 0.0001. (**B**,**D**) Brightfield images of toluidine blue (TB) staining specific for GAGs, on cells seeded with CCM. Scale bar = 200 µm.

In the absence of osteogenic signals, all studied cell groups showed an enhancement in GAG production when exposed to CCM (Figure 12A,C). This GAG expression pattern was also confirmed by TB staining (Figure 12B,D).

Furthermore, and as expected, the combination of a cartilaginous ECM, such as the one provided by the suture-mimicking scaffolds, and the exposure to CCM, caused a significant increase in the expression of GAGs, compared to the GAG production in cells seeded on bone-mimicking scaffolds.

As described in the previous section (Figure 11 and Figure 12) an increase in the expression of chondrogenic markers likely reflects the combined influence of the scaffold architecture and the biochemical features of the surrounding microenvironment, such as ECM and media composition. While this microenvironment recapitulates features associated with a non-pathological niche, it should not be interpreted as directly promoting suture patency. Rather, it appears to support the maintenance of a chondrogenic-like phenotype. Our results confirmed once again the key role of the biochemical and biophysical properties of the surrounding microenvironment in the differentiation patterns of the different cell population from the calvarial sutures.

## 4. Discussion

Calvarial bone formation and suture development is a highly regulated process that can be altered in developmental diseases, such as craniosynostosis, where an accelerated ossification of the patent sutures of the skull prematurely lead to their fusion. Previous studies focused on evaluating the effect of these alterations in the pathogenesis of craniosynostosis have demonstrated an abnormal interaction between cells and their ECM, suggesting a role for an altered mechanotransduction mechanism in defining the timing of calvarial suture fusion [7,9,13]. For sutures to function as major sites of intramembranous bone expansion, sufficient progenitor cells need to differentiate into new bone cells to be recruited into the edges of the bone fronts, while ensuring that the cells within the suture remain undifferentiated [8,9,10]. We postulated that biochemical and biophysical alterations in the extracellular microenvironment may activate bone differentiation signaling cascades and alter osteogenic commitment of the undifferentiated cells within the calvarial sutures. A limitation of the present model is the absence of signals derived from dural cells or the effect of physiological mechanical loading, both of which are known to regulate suture patency and osteogenesis. Therefore, our findings should be interpreted within the context of a controlled microenvironment designed to emulate the selected biophysical and biochemical properties of the native ECM of the human skull.

To test our hypothesis, we first characterized the cell populations isolated from the freshly resected calvarial tissues of children diagnosed with NS-CS. By investigating the native stemness and lineage-specificity of cells isolated from calvarial bone and patent and fused sutures, we found that cells from patent sutures exhibited the highest degree of multilineage plasticity (Figure 2). Furthermore, flow cytometry-based characterization demonstrated that cells from patent sutures also conserve the strongest mesenchymal progenitor properties with the lowest proportion of cells positive for hematopoietic and immune markers. This is consistent with the biology of the intrasutural mesenchyme, which is enriched in mesenchymal progenitor cells and lacks the hematopoietic component typical of diploic bone, as evidenced by our histological findings. Finally, genetic profiling by PCR confirmed significant upregulation of markers targeting neural crest-derived cells and multipotency on cells from patent sutures (Figure 2). The distinct embryological origins of craniofacial structures may further explain these differences in cellular potential. While calvarial bones arise from progenitor cells of the paraxial mesoderm, the majority of calvarial suture mesenchyme derives from neural crest-origin cells, which are known to retain higher developmental plasticity and self-renewal potential [34,35,36]. This neural crest-derived population may thus endow the patent suture with a greater regenerative and adaptive capacity, allowing it to respond dynamically to mechanical and biochemical cues that regulate bone formation and suture patency. Additionally, the use of samples derived from different suture types (e.g., coronal and sagittal sutures) may represent a source of variability, as differences in embryological origin and developmental characteristics could have influenced the observed cellular behavior. In contrast, cells isolated from prematurely fused sutures exhibited reduced multipotency, consistent with a more committed osteogenic phenotype and a loss of mesenchymal progenitor cell-like features (Figure 2).

Our findings together do not only offer the initial characterization of the basal differences between cell populations isolated from calvarial bone and patent and fused sutures, but also confirm that cells from patent sutures of children diagnosed with NS-CS still conserve their native lineage-specificity and have the higher multilineage differentiation compared to the other sample groups (Figure 2). These results are in line with those reported by Isern and collaborators who described that both the neural crest and the mesoderm are a source of mesenchymal progenitor populations and osteolineage cells which have distinct functions and contributions to the development of different types of tissue in the body, from the bones to the brain tissue [34]. Proliferative mesoderm-derived cells participate in fetal skeletogenesis and lose their native stemness soon after birth while quiescent neural crest-derived cells preserve their multilineage plasticity. It has also been suggested that while mesoderm-derived progenitors give rise to bone and cartilage, the neuroectoderm provides an additional source of mesenchymal progenitor cells, which are endowed with specific multilineage functions [35].

In particular, we found a major cell population expressing high levels of the multipotency marker CD106 among our cells isolated from patent sutures, while a study developed by Zhao and collaborators identified Gli1+ progenitor-like cells as one of the main cell populations in craniofacial bones, which were, in addition, described as responsible for maintaining calvarial suture patency [35]. Similarly, research conducted by Maruyama and collaborators not only identified Gli1+ progenitor-like cells but also a different population which expresses high levels of Axin2 and the location of which is restricted to the midline of the suture. These Axin2+ progenitor-like cells exhibited long-term self-renewing, clonal expanding and differentiating abilities and it was suggested that a reduction in their number resulted in the premature fusion of the calvarial sutures [36]. However, their developmental relationships remain unclear and suture cell populations have yet to be fully characterized and identified [34]. Taken together, the premature fusion of the sutures in NS-CS may not only be associated with changes in the biophysical stimuli provided by their surrounding extracellular microenvironment, but also to the inherent functional capacity of the progenitor cells within the calvarial sutures to sense and respond to biochemical stimuli.

In order to establish the role that the ECM plays in the pathophysiology of craniosynostosis, we analyzed and defined the native components of the in vivo tissue microenvironment of the calvarial bone and sutures. The identification of key structural components within the calvarial niche provided the foundational framework for the rational design and development of our 3D patient-based model of craniosynostosis. For this purpose, we histologically examined freshly resected tissues by using histological staining. H&E and toluidine blue staining showed a dense appearance, characteristic of compact bone, in samples from calvarial bone, while sections from patent sutures were composed of a denser fibrillar tissue with a positive expression of GAGs (Figure 3), characteristic of cartilage. Interestingly, tissues from fused sutures showed an intermediate stage (Figure 3). The histological findings of a denser, compact bone-like tissue in fused sutures and GAG-rich fibrillar ECM in patent sutures are consistent with prior reports describing the fibrocartilaginous character of patent calvarial sutures and the mineralized phenotype of fused regions [9,37,38]. However, the exact roles of proteoglycans and GAGs in calvarial development still require further investigation [38]. In addition, strong ALP expressions and high calcium deposition were found in the prematurely fused sections compared to patent sutures (Figure 4). However, ALP activity was greater than the ECM mineralization patterns (Figure 4), which might indicate that fused suture sections are still in the early stage of ossification. Elevated ALP activity and increased calcium deposition in fused sutures have also been reported in experimental animal models of premature suture fusion, while an increased ALP activity relative to low ECM mineralization supports the notion that these fused regions remain in an early ossification stage [39,40].

Once the native microenvironment of the calvarial tissues was characterized, we established the differentiation potential of the three different sample groups into an osteogenic phenotype, while considering cells isolated from the calvarial bone as fully differentiated osteoblasts. Findings showed similar high ALP activity by cells from fused sutures and calvarial bone when cultured in growth media (Figure 5A). In contrast, levels of mineralization remained consistently low throughout the sample groups during the three different evaluated time points (Figure 5B). When samples were cultured in osteogenic media, cells from fused sutures presented significantly higher ALP activity and higher calcium release than cells from patent sutures (Figure 5A,B), reaching the peak of expression after 14 and 21 days in culture, respectively, in 2D. These results indicated that within an osteogenic surrounding microenvironment, cells from fused sutures differentiate rapidly towards an osteogenic phenotype when compared to patent sutures. Previous analyses carried out by Regelsberger and co-authors at the suture cellular level suggested that the mineralized segments in fused versus patent sutures are associated with different stages in the course of a normal osteogenesis process. Bony edges in prematurely fused sutures revealed key structural and morphological characteristics indicative of a more advanced stage in bone development and maturation, without any signs of a pathological or defective osteogenesis [41]. Therefore, this data supported the theory of a normal ossification process in fused sutures, with osteogenesis mechanisms simply being activated prematurely.

Finally, we emulated the physical and biological properties of the native ECM of the human skull. Nowadays, 3D in vitro cell culture models have gained considerable attention as a powerful research tool that more faithfully recapitulates the in vivo microenvironment of native tissues compared to conventional 2D monolayer culture systems [39,40]. Our patient-derived 3D model incorporates key biochemical and biophysical characteristics of calvarial tissues, providing a more physiologically relevant environment than conventional 2D cultures. Consequently, it offers a valuable platform for investigating how ECM cues influence cellular behavior and contribute to the mechanisms underlying premature suture fusion.

We built a 3D patient-based culture system composed of two different types of scaffolds, bone-mimicking scaffolds and cartilage-mimicking scaffolds, the latter one imitating the cartilage-like tissue characteristic of calvarial sutures. While previous characterization carried out in our lab confirmed that the overall scaffold porosity remained unchanged [27], SEM imaging demonstrated differences in pore size between the two different scaffold groups (Figure 6). Cells cultured within these 3D microenvironments showed differential expression of osteogenic and chondrogenic markers, as well as differences in ECM mineralization patterns (Figure 7), as confirmed by Alizarin Red staining (Figure 8). Unlike conventional 2D systems—which often fail to reproduce the spatial organization, mechanical cues and cell–matrix interactions—our 3D culture platforms enabled the assessment of cellular response within a spatially organized scaffold environment containing distinct ECM components. Using both 2D and 3D culture systems, we assessed the differentiation potential of the different sample groups under osteogenic and chondrogenic microenvironmental conditions. Cells isolated from fused sutures consistently exhibited higher expression of osteogenic markers and greater GAG production under the specific scaffold and culture conditions (Figure 9 and Figure 10). Trends in ALP activity and calcium deposition were further supported by histological analysis (Figure 11). Although physiological calvarial suture closure is primarily associated with intramembranous ossification [42], this combined osteogenic and chondrogenic response may reflect the involvement of endochondral-like ossification pathways in the pathological suture fusion in NS-CS. However, further studies will be required to clarify the mechanisms underlying these observations.

In a recent study carried out by Bok and collaborators, they established the presence of two distinct progenitor cell lineages in the calvarial sutures, with both populations contributing to physiological calvarial mineralization. One of these groups was classified as cathepsin K (CTSK) lineage and described as specialized for intramembranous bone formation. The second group is a discoidin domain-containing receptor 2 (DDR2) lineage that characteristically mediates endochondral ossification without marrow formation. This publication indicated that a third fundamental form of bone formation, endochondral ossification without haematopoiesis, might exist alongside traditional intramembranous and endochondral ossification within the calvarial sutures, supporting the results found with our 3D culture system [43].

Interestingly, the determination of the intrinsic differentiation capacity of the studied cell populations showed that exposure to CCM, TGF-β3-rich culture media, caused a significant increase in the expression of GAGs, while removal of TGF-β3 significantly increased ALP activity and calcium deposition (Figure 10 and Figure 11). These results align with those reported in a study on a fetal calvarial culture model carried out by Opperman and collaborators where they demonstrated that removal of TGF-β3 activity induced premature suture fusion. Data presented showed that suture closure induced by the removal of TGF-β3 activity was preceded by elevated levels of cell proliferation and differentiation markers, while the addition of exogenous TGF-β3 to cultured calvarial suture prevented osteogenic cell differentiation [44]. Findings demonstrated that cells from fused sutures are able to reach a more mature differentiated phenotype faster than cells from patent sutures. More importantly, this increased response significantly depended on the osteo- and/or chondroinductive biochemical signals present in the surrounding microenvironment. While the previous literature has largely described craniosynostosis from a clinical and genetic perspective, Homayounfar and collaborators [45] reported distinct gene expression signatures associated with the embryonic origin of calvarial tissues. Our findings extended these observations by demonstrating that microenvironmental cues alone can modulate osteogenic commitment, further supporting the multifactorial nature of the disease.

In conclusion, this study identified differences in responsiveness between the cell populations isolated from calvarial bone and patent and fused sutures when exposed to variations in biochemical and biophysical signals. This adds weight to the growing evidence that NS-CS is linked to an abnormal biochemical and biophysical environment, confirming a microenvironment-dependent accelerated bone formation in cells from fused sutures (Figure 7, Figure 8, Figure 9, Figure 10 and Figure 11). In addition to providing an insight into the pathology of the premature ossification of NS-CS, described as an endochondral ossification process activated by an abnormal surrounding microenvironment, these findings showed the effect of biochemical and biophysical alterations on patency and ossification of the different sample groups. Furthermore, by demonstrating that the activation of osteogenic activity in cells from fused sutures is strongly modulated by microenvironmental cues, this model may serve as a platform for the development and testing of therapeutic strategies aimed at modulating ECM properties or associated signaling pathways. Such approaches could contribute to restore a non-pathological microenvironment, regulating aberrant osteogenic differentiation and ultimately preventing or delaying premature suture fusion. Finally, this study generated an in vitro 3D model of NS-CS which helped to uncover the mechanotransducive mechanisms and endochondral pathways underlying NS-CS, paving the way for development of new therapeutic strategies to address prematurely fused sutures.

## Data Availability

The data presented in this study are available on request from the corresponding author due to ethical restriction.
